# Pre-sensitization of Malignant B Cells Through Venetoclax Significantly Improves the Cytotoxic Efficacy of CD19.CAR-T Cells

**DOI:** 10.3389/fimmu.2020.608167

**Published:** 2020-12-09

**Authors:** Mingya Yang, Lei Wang, Ming Ni, Brigitte Neuber, Sanmei Wang, Wenjie Gong, Tim Sauer, Leopold Sellner, Maria-Luisa Schubert, Angela Hückelhoven-Krauss, Jian Hong, Lixin Zhu, Christian Kleist, Volker Eckstein, Carsten Müller-Tidow, Peter Dreger, Michael Schmitt, Anita Schmitt

**Affiliations:** ^1^Department of Internal Medicine V, University Clinic Heidelberg, Heidelberg University, Heidelberg, Germany; ^2^Department of Hematology, The First Affiliated Hospital of Anhui Medical University, Anhui, China; ^3^Department of Hematology, The Affiliated Hospital of Guizhou Medical University, Guizhou, China; ^4^Department of Hematology, The First Affiliated Hospital of Nanjing Medical University, Collaborative Innovation Center for Cancer Personalized Medicine, Nanjing, China; ^5^Department of Hematology, The First Affiliated Hospital of Soochow University, Suzhou, China; ^6^Oncology Business Unit-Medical Affairs, Takeda Pharma Vertrieb GmbH & Co. KG, Berlin, Germany; ^7^Department of General Surgery and Central Laboratory, The First Affiliated Hospital of Anhui Medical University, Anhui, China; ^8^Department of Nuclear Medicine, University Clinic Heidelberg, Heidelberg University, Heidelberg, Germany; ^9^National Center for Tumor Diseases (NCT), German Cancer Consortium (DKTK), Heidelberg, Germany

**Keywords:** CD19.CAR-T cells, BH3 mimetics, venetoclax (ABT-199), S63845, apoptosis

## Abstract

Chimeric antigen receptor (CAR) T cell therapy has shown promising responses in patients with refractory or relapsed aggressive B-cell malignancies that are resistant to conventional chemotherapy or stem cell transplantation. A potentially combinatorial therapeutic strategy may be the inhibition of anti-apoptotic Bcl-2 family proteins, overexpressed in most cancer cells. In this study we investigated the combination of 3rd-generation CD19.CAR-T cells and the BH3 mimetics venetoclax, a Bcl-2 inhibitor, or S63845, a Mcl-1 inhibitor, under three different treatment conditions: pre-sensitization of cancer cells with BH3 mimetics followed by CAR-T cell treatment, simultaneous combination therapy, and the administration of BH3 mimetics after CAR-T cell treatment. Our results showed that administration of CAR-T cells and BH3 mimetics had a significant effect on the quantity and quality of CD19.CAR-T cells. The administration of BH3 mimetics prior to CAR-T cell therapy exerted an enhanced cytotoxic efficacy by upregulating the CD19 expression and pro-apoptotic proteins in highly sensitive tumor cells, and thereby improving both CD19.CAR-T cell cytotoxicity and persistence. In simultaneous and post-treatment approaches, however, the quantity of CAR-T cells was adversely affected. Our findings indicate pre-sensitization of highly sensitive tumor cells with BH3 mimetics could enhance the cytotoxic efficacy of CAR-T cell treatment.

## Introduction

Chimeric antigen receptor (CAR) modified T cells have revolutionized the field of immunotherapy for cancer (1). CD19.CAR-T cells induced clinical responses in more than 60% of patients with relapsed/refractory (r/r) B-cell acute lymphoblastic leukemia (ALL) (2). However, approximately 10–20% of patients fail to enter remission after receiving CD19.CAR-T cells (3). Of note, it has less clinical efficacy in patients with chronic lymphocytic leukemia (CLL) and large B cell non-Hodgkin’s lymphoma (NHL) (4). Therefore, there is a strong medical need to improve the efficacy of CD19.CAR-T cell therapy.

Several strategies, from defining the best starting material, developing novel CAR constructs and improving the manufacturing process up to combining CAR-T cell therapy with other anti-tumor agents, are intensively studied to optimize the clinical efficacy of CAR-T cell therapy (3, 5). Especially, the combinatorial approach is to target cancer cells with a “dual-hit therapy” by improving the efficiency of CAR-T cells while eliminating immunosuppressive cells. Subsequently, this concept has been proven by combining CAR-T cells with radiotherapy, immune-checkpoint inhibitors, or tyrosine kinase inhibitors (6–8).

A characteristic of CLL and NHL is the overexpression of anti-apoptotic Bcl-2 family proteins. Hence, the inhibition of the anti-apoptotic machinery of cancer cells as a promising therapeutic option has driven the development of compounds that antagonize the function of anti-apoptotic proteins termed BH3 mimetics, which have shown meanwhile remarkable efficacy in clinical studies (9). Thus, combination of CAR-T cells with BH3 mimetics might constitute a promising therapeutic strategy.

In our study, two BH3 mimetic compounds venetoclax, a Bcl-2 selective inhibitor, and S63845, a Mcl-1 selective inhibitor, were combined with CAR-T cells. The cytotoxic efficacy was evaluated in pre-treatment, simultaneous and post-treatment systems.

## Materials and methods

### Cell Lines

U698M, Daudi, 380, Nalm6, K562, and MeC2 cell lines used in this study were purchased from German Collection of Microorganisms and Cell Cultures (DSMZ). The culture condition and cell maintaining for each cell line were according to official guidelines of the DSMZ. All cell lines were used in logarithmic growth phase and mycoplasma were excluded by polymerase chain reaction (PCR).

### Cell Preparation

#### Isolation of Peripheral Blood Mononuclear Cells

Primary tumor cells were derived from ten newly diagnosed, untreated B-CLL patients who have signed informed consent at Heidelberg University Hospital. Buffy Coats from consenting healthy donors (HDs) were obtained from the Heidelberg Blood Bank. Peripheral blood mononuclear cells (PBMCs) were separated by a ficoll gradient, and then frozen in liquid nitrogen. Sample collection and analysis were approved by the Ethics Committee of the University of Heidelberg (S-254/2016).

### Dead Cell Removal

Dead cells were excluded by EasySep™ dead cell removal kit (STEMCELL Technologies) according to manufacture instruction. Untouched living cells were used for further experiments.

### Manufacturing of 3rd-Generation CD19.CAR-T Cells

Manufacture of CD19.CAR-T cells mainly consisted of retroviral vector (SFG CD19.CD28/4-1BB/ζ) production, T cell transduction and CAR-T cell expansion. The retroviral vector used in this study was produced at Baylor College of Medicine in Houston (10). The generation of CD19.CAR-T cells followed our standard operating procedure (SOPs) as previously described (11). CD19.CAR-T cells were harvest on day 10 for following experiments.

### Quantitative Real-Time PCR (qPCR)

Total RNA was isolated with Trizol^®^ Reagent (Thermo Fisher Scientific), followed by removal of genomic DNA. Complementary DNA (cDNA) was reverse transcribed using the PrimeScript™ RT Reagent Kit (TAKARA). q-PCR was carried out with SYBR Green master mix (TAKARA) on StepOnePlus Real-Time PCR system. The expression of Mcl-1 and Bcl-2 was determined by Mcl-1 primers and Bcl-2 primers, respectively. The gene expression was normalized to GAPDH. The list of used primers is provided in **Supplementary Table 1**. Samples were run in triplicate. StepOne™ software v2.3 was used for analysis.

### Western Blot

Dead cells were removed by magnetic beads sorting. Living cells were used for further protein extraction. Total protein was isolated using radioimmunoprecipitation buffer (RIPA) (Thermo Fisher Scientific). Samples mixed with loading buffer (Sigma-Aldrich) were heated to 95°C for 5 min to denature the protein. Each sample was electrophoresed on NuPAGE 4–12% bis-tris gel (Invitrogen™) and transferred to nitrocellulose membrane (Carl Roth). Membranes were incubated at 4°C overnight with the following primary antibodies: anti-human Mcl-1 (abcm), anti-human Bcl-2 (abcm), anti-human Bcl-xl antibody (abcm), anti-human BAK antibody (abcm), and anti-human β-actin antibody (Santa Cruz). The blots were then probed with HRP-linked anti-rabbit IgG antibody (cell signaling) and anti-mouse IgG antibody (cell signaling). The reactions were developed using the ECL™ prime western blotting detection reagent (GE Healthcare). Membranes were imaged on FUSION imaging system (GE Healthcare), and band intensities were quantified with ImageJ program.

### CellTiter-Glo luminescent cell viability assay

To test the activity and sensitivity of Mcl-1 inhibitor S63845 and Bcl-2 inhibitor venetoclax (Medchem express), cells were seeded into 384-well plates in the presence of 10-point 1:3 serial dilutions of compounds starting from 10 μM. Luminescence was measured after 48-h co-culture using the CellTiter Glo reagent (Promega). Results were normalized to the values of cells that had been treated with 0.1% PBS (vehicle) in culture medium for 48 h.

### *In Vitro* Treatment Systems

To assess the effect of inhibitors on both CD19.CAR-T cells and tumor cells, three different co-culture systems including pre-, simultaneous, and post-treatment system, were established. The number of residual tumor cells and T cells in cultures were quantified every five days using flow cytometry. Subsequently, CD19.CAR-T cells were re-challenged with the identical number of fresh tumor cells until no or only a few CAR-T cells were left in the co-culture systems.

### Pre-treatment System

The tumor cells were pre-treated by venetoclax or S63845 in a series of concentrations for 24 h. After dead cell removal and washing out the BH3 mimetics, 6 × 10^4^ venetoclax pre-treated 380 cells and 3 × 10^3^ S63845 pre-treated U698M cells were introduced into the treatment system and co-cultured with 1.5 × 10^4^ CD19.CAR-T cells.

### Simultaneous Treatment System

1.5 × 10^4^ CD19.CAR-T cells were co-cultured with Daudi cells at an E:T ratio of 1:1 in the absence or presence of different concentrations of venetoclax or S63845. When CAR-T cells were re-challenged with fresh tumor cells, the same amount of BH3 mimetics was added as well.

### Post-treatment System

After 24-h co-culture of CD19.CAR-T cells with Daudi cells, different concentrations of BH3 mimetics were introduced to the system. Subsequently, the same amount of BH3 mimetics was always added after 24-h re-challenge by fresh tumor cells.

### Flow Cytometry

Marker expression was evaluated by multicolor flow cytometry. Cells were harvested and stained with Near-IR, followed by incubation with different combinations of antibodies, as shown in **Supplementary Table 2**. The acquisition was performed on an LSR II device (BD biosciences). BD FACSDiva software (BD biosciences) was used for the data analysis.

### Transduction Efficiency

Following Near-IR staining, CAR-T cells from day 7 were stained with antibody cocktail for 30 min at room temperature (RT) in the dark, then fixed using fixation buffer (R&D system) before acquisition.

### Activation Marker Staining

After 24-h stimulation by Daudi cells at an E:T ratio of 1:1 in the absence or presence of different concentrations of BH3 mimetics, CD19.CAR-T cells were harvested and stained with activation marker in addition with other surface maker antibodies for 30 min at RT in the dark.

### Cell Viability

Near-IR and Annexin V FITC were used to check the cell viability. After Near-IR and surface marker staining, cells were re-suspended in Annexin V binding buffer and incubated with Annexin V for 15 min at RT in the dark. Then cells were washed and re-suspended. Acquisition was performed immediately after adding 50 μl Counting Beads (Thermo Fisher Scientific). 5,000 counting beads were acquired for each sample.

### Anti-apoptotic Bcl-2 Family Protein Staining

Cells from 24-h co-culture with Daudi cells (E:T = 1:1) in the absence or presence of BH3 mimetics were stained with Near-IR, followed by fixation and permeabilization. Afterwards, cells were washed and stained with antibody cocktails consisting of surface marker and anti-apoptotic Bcl-2 family antibodies for 30 min at RT in the dark.

### Intracellular Cytokine Staining

Following Near-IR staining, activated CD19.CAR-T cells, which were stimulated with Daudi cells in the presence of monensin (Invitrogen) and brefeldin A (Invitrogen), were fixed and permeabilized, then finally stained for 30 min with surface marker and cytokine antibodies at RT in the dark.

### Cell Quantification

Cells were harvested and stained with Near-IR to exclude dead cells, followed by staining specific tumor markers in addition with other surface maker antibodies for 30 min at RT in the dark. Acquisition was performed immediately after adding 50 μl Counting Beads (Thermo Fisher Scientific). 10,000 counting beads were acquired for each sample.

### Exhaustion Marker Staining

Following Near-IR staining, CD19.CAR-T cells were stained with exhaustion markers in addition with other surface maker antibodies for 30 min at RT in the dark.

### Calcein AM Assay

The potency of CD19.CAR-T cells was determined by a standard Calcein AM assay. Briefly, living tumor cells were labeled with Calceim AM reagent. Then CD19.CAR-T cells were plated in triplicate with either 5 × 10^3^ BH3 mimetics pretreated living tumor cells or 5 × 10^3^ target tumor cells in the absence or presence of different concentrations of BH3 mimetics, using effector to target (E:T) ratio of 30:1. After a 3-h co-culture, supernatants were harvested, and fluorescence of Calceim AM was measured by a PerkinElmer plate reader. The maximal release and spontaneous release were determined by culturing the target cells with 2% Triton X-100 (Sigma-Aldrich) in PBSS and PBSS alone, respectively. The percentage of lysis was calculated following the formula: [experimental release - spontaneous release]/[maximal release - spontaneous release] × 100.

### Statistical Analysis

Statistical analysis was performed using IBM SPSS 22 for Windows and GraphPad Prism 8.0. The IC_50_ values were calculated using nonlinear regression algorithms in IBM SPSS v22. The difference among concentrations of inhibitors was calculated using Tukey’s multiple comparisons test in GraphPad Prism 8.0 and paired t-test in IBM SPSS v22. In all tests, a *p* value < 0.05 was considered to be statistically significant.

## Results

### Cytotoxic Activity of Venetoclax and S63845

The cytotoxic activity of venetoclax and S63845 was tested in a panel of six leukemia and lymphoma cell lines using the CellTiter Glo assay. The cell density used in this assay could ensure the cell growth rate to reach the exponential phase after 48 h of culture (**Supplementary Figure 1**).

**Figure 1:**
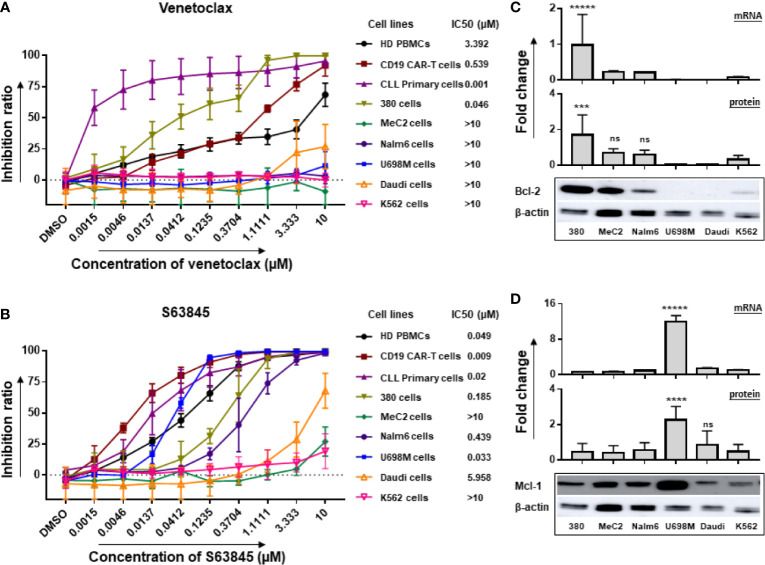
Inhibitory effect of venetoclax and S63845 on tumor cell lines associated with mRNA and protein levels of Bcl-2 and Mcl-1. The inhibition ratios of the Bcl-2 selective inhibitor venetoclax **(A)** and Mcl-1 specific inhibitor S63845 **(B)** on different cells after 24-h incubation, which were calculated as the following formula: inhibition ratio = 100 – (experimental luminescence/PBS luminescence) × 100. IC_50_ was determined by IBM SPSS v22, regression-probit. Three different individual donors have been analyzed, besides CLL primary cells from ten newly diagnosed and untreated patients. 380 cells and U698M cells showed a high sensitivity to venetoclax and S63845, respectively. The mRNA and protein expression of Bcl-2 **(C)** and Mcl-1 **(D)** in six tumor cell lines were normalized to GAPDH and β-actin, respectively. Protein bands were analyzed by ImageJ program. Each experiment contains three replicates. The statistical analysis was performed using a Tukey’s multiple comparisons test in GraphPad Prism 8.0. A *p* < 0.05 was considered to be statistically significant (*). ns means no significant difference. The number of asterisks means the number of significant differences among the comparison. ns means no significant difference. 380 had highest RNA and protein levels of Bcl2 expression and U698M showed highest Mcl-1 expression in both RNA and protein levels.

One out of six tested cell lines (380 cells) showed a high sensitivity to venetoclax (IC_50_ < 0.1 µM), and five cell lines (MeC2, Nalm6, U698M, Daudi and K562 cells) were low sensitive (IC_50_ > 1 µM) (**Figure 1A**). Similarly, one cell line (U698M cells) was highly sensitive (IC_50_ < 0.1 µM) to S63845, two (380 and Nalm6 cells) were moderately sensitive (0.1 µM < IC_50_ < 1 µM) and three (MeC2, Daudi and K562 cells) were low sensitive (IC_50_ > 1 µM) (**Figure 1B**). Of note, all primary cells derived from ten newly diagnosed and untreated CLL patients were highly sensitive to both venetoclax and S63845. IC_50_ values were in the low-nanomolar range (**Figures 1A, B**). Both BH3 mimetics exerted dose-dependent cytotoxic activity (**Figures 1A, B**), which strongly connected to Bcl-2 and Mcl-1 expression in these tumor cells showing high expression of Bcl-2 or Mcl-1 on both RNA and protein levels (**Figures 1C, D**). However, PBMCs from HD and CD19.CAR-T cells displayed also a high sensitivity to venetoclax (IC_50_ of PBMCs: 3.392 µM; IC_50_ of CD19.CAR-T cells: 0.539 µM) and S63845 (IC_50_ of PBMCs: 0.049 µM; IC_50_ of CD19.CAR-T cells: 0.009 µM).

### Increase of Killing Efficiency of CD19.CAR-T Cells by Pre-treatment of Highly Sensitive Tumor Cell Lines With BH3 Mimetics

The CD19 CAR molecule could be efficiently transduced on T cells (78.9% ± 7.32) (**Supplementary Figures 2A, B**) with a high viability (84.69% ± 4.02) and 10-fold expansion (*p* < 0.05) (**Supplementary Figures 2C, D**). The potency and specificity of killing capacity of CD19.CAR-T cells were assessed by a standard Calcein AM assay, showing high cytotoxicity against CD19^+^ tumor cells but negligible activity against CD19^-^ K562 cells (**Supplementary Figure 2E**). Strikingly, the density of CD19 antigen on tumor cells influenced the killing efficiency (**Supplementary Figure 2F**). Taken together, the results suggest that our CD19.CAR-T cells exhibit a potent anti-tumor effect *in vitro*.

**Figure 2:**
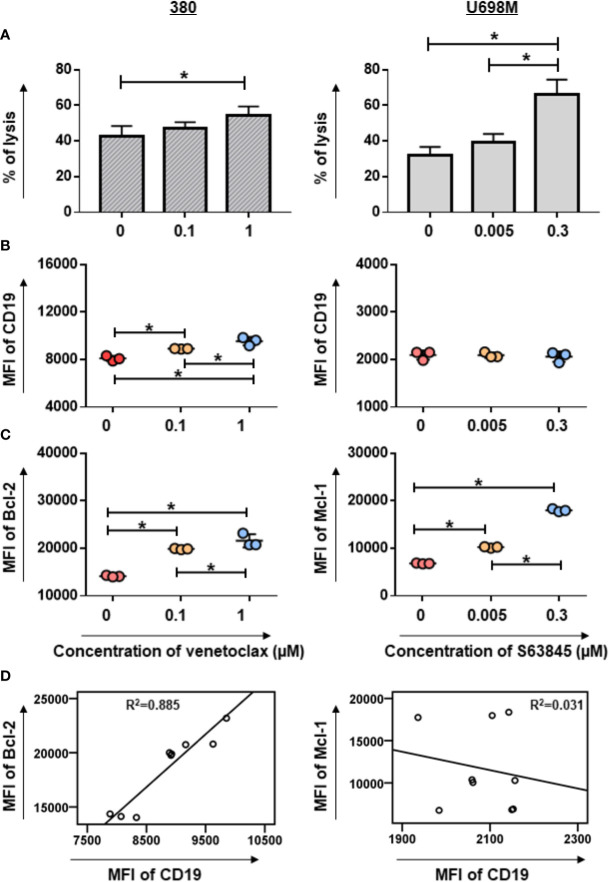
Pre-sensitization of tumor cell lines by venetoclax or S63845 increased the cytotoxic efficiency of CD19.CAR-T cells. The killing efficiency of CD19.CAR-T cells against venetoclax pre-treated 380 cells (A, left panel) and S63845 pre-treated U698M cells (**A**, right panel) determined by Calcein AM assay. The percentage of lysis was calculated as followed: [experimental release – spontaneous release]/[maximum release – spontaneous release] × 100. The killing efficiency of CD19.CAR-T cells was enhanced, when the highly sensitive tumor cells were pre-treated by BH3 mimetics. The CD19 expression on 380 cells (**B**, left panel) could be up-regulated, following by pre-treatment of venetoclax for 24h, but not on pre-treated U698M cells (**B**, right panel). The MFI of Bcl-2 and Mcl-1 expression on venetoclax pre-treated 380 cells (**C**, left panel) and on S63845 pre-treated U698M cells (**C**, right panel) were increased, respectively. The correlations of Bcl-2 with CD19 expression in venetoclax pre-treated 380 cells (**D**, left panel) and Mcl-1 with CD19 expression in S63845 pre-treated U698M cells (**D**, right panel) were analyzed using IBM SPSS v22. It showed that CD19 expression is highly associated with Bcl-2 expression. The pre-treatment duration was 24-h. Three different individual donors have been analyzed. Each experiment was performed in triplicate. For statistical analysis a Tukey**’**s multiple comparisons test was used in GraphPad Prism 8.0. A *p* < 0.05 was considered to be statistically significant (*).

To evaluate the cytotoxic capacity of CD19.CAR-T cells on BH3 mimetic pre-treated tumor cells, BH3 mimetic highly sensitive and low sensitive tumor cells were cultured with BH3 mimetics for 24 h prior to the addition of CD19.CAR-T cells. Notably, we observed a dramatically increased killing efficiency of CD19.CAR-T cells when S63845 pre-treated, highly sensitive U698M cells were used as target cells, and a modest increase of killing efficiency of CD19.CAR-T cells on venetoclax pre-treated 380 cells (**Figure 2A**). However, this improved cytotoxic effect was not observed on BH3 mimetics pre-treated low sensitive Daudi cells for both venetoclax and S63845 (**Supplementary Figure 3**).

### Up-regulation of Expression of CD19 Antigen, Anti-apoptotic, and Pro-apoptotic Proteins by Pre-treatment of Highly Sensitive Tumor Cell Lines With BH3 Mimetics

To characterize the mechanism of enhanced killing efficiency, we evaluated changes in the expression of CD19 antigen, anti-apoptotic and pro-apoptotic Bcl-2 family proteins in BH3 mimetics-pretreated tumor cells. Following exposure of 380 cells to venetoclax for 24 h, a considerable increase of CD19 expression was observed in pre-treated 380 cells, whereas S63845 pre-treated U698M cells showed no substantial changes (**Figure 2B**). Furthermore, pre-treatment of tumor cells with BH3 mimetics resulted in an up-regulation of Bcl-2 and Mcl-1 expression in venetoclax highly sensitive 380 cells and S63845 highly sensitive U698M cells (**Figure 2C**), respectively.

Of note, the expression of CD19 was highly associated with Bcl-2 expression in venetoclax pre-treated 380 cells (R^2^ = 0.885, *p* = 0.000, **Figure 2D**). The co-expression of CD19 and Bcl-2 was further confirmed by a predictive analysis using GeneMANIA database (12) (**Supplementary Figure 4A**). However, there was no correlated expression between CD19 and Mcl-1 in S63845 pre-treated U698M cells (R^2^ = 0.031, *p* = 0.652, **Figure 2D** and **Supplementary Figure 4B**).

**Figure 3:**
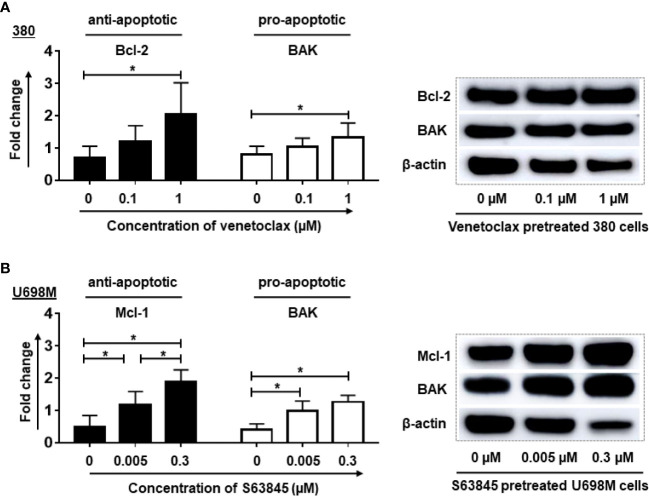
Pre-treatment of tumor cells by BH3 mimetics increased the expression of anti-apoptotic and pro-apoptotic proteins in tumor cells. After the pre-treatment, the expression of Bcl-2 and BAK proteins in venetoclax pre-treated 380 cells **(A)** as well as Mcl-1 and BAK in S63845 pre-treated U698M cells **(B)** were increased. The treatment duration was 24-hr. Dead cells were removed by magnetic beads sorting followed by protein extraction. Three different individual experiments have been analyzed. β-actin was used as a loading control to normalize the levels of target proteins. Protein bands were analyzed by ImageJ program. Each experiment was performed in duplicates. For statistical analysis, a Tukey**’**s multiple comparisons test was used in GraphPad Prism 8.0. A *p* < 0.05 was considered to be statistically significant (*).

Consistently with the flow cytometry results, western blot analysis showed a significant elevation of anti-apoptotic protein Bcl-2 accompanied by a significant increase of pro-apoptotic protein BAK in venetoclax pretreated 380 cells (**Figure 3A**). Similarly, S63845 pretreated U698M cells increased not only the expression of anti-apoptotic protein Mcl-1, but also the pro-apoptotic protein BAK (**Figure 3B**).

Altogether, our results indicate that the enhancement of killing efficiency of CD19.CAR-T cells tightly correlated with the increased expression of CD19 antigen, anti-apoptotic and pro-apoptotic Bcl-2 family proteins in inhibitor highly sensitive tumor cells.

### Increase of Anti-tumor Activity by Simultaneous Treatment With CD19.CAR-T Cells and BH3 Mimetics

**Figure 4:**
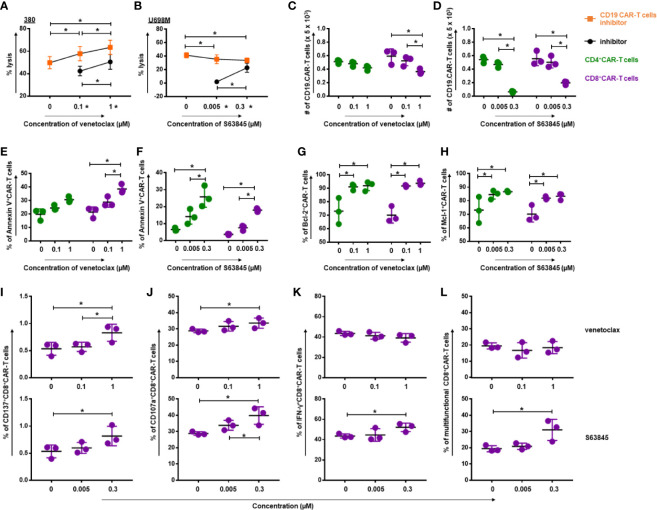
Simultaneous treatment approach increased the quality of CD19.CAR-T cells but decreased the quantity. The killing efficiency of CD19.CAR-T cells against 380 cells (E:T = 30:1) in the presence of venetoclax **(A)** and against U698M cells (E:T = 30:1) in the presence of S63845 **(B)** was determined by Calcein AM assay. It shows a better antitumor activity, when CD19.CAR-T cells were combined with venetoclax **(A)**, but not with S63845 **(B)**. Quantification of living CD4^+^ CAR-T cells and CD8^+^ CAR-T cells was determined by flow cytometry, showing a decrease of CD8^+^CD19.CAR-T cells after 24-h co-culture with 380 cells in the presence of venetoclax **(C)** and a reduction of both CD4^+^ and CD8^+^CD19.CAR-T cells after 24-h co-culture with U698M cells in the presence of S63845 **(D)**. The percentages of apoptotic CD4^+^ and CD8^+^ CAR-T cells were increased after 24-h co-culture with 380 cells in the presence of venetoclax **(E)** or U698M cells in the presence of S63845 **(F)**. The percentage of both Bcl-2^+^CD4^+^ and Bcl-2^+^CD8^+^ CAR-T cells were elevated after 24-h co-cultured with 380 cells in the presence of venetoclax **(G)**. An up-regulation of Mcl-1^+^CD4^+^ and Mcl-1^+^CD8^+^ CAR-T cells was observed after 24-h co-cultured with U698M cells in the presence of S63845 **(H)**. Increased expression of activation marker CD137 on CD8^+^ CAR-T cells was detected using flow cytometry after 24-h stimulation by 380 cells in presence of venetoclax (**I**, upper panel) and S63845 (**I**, lower panel). The influence of venetoclax (upper panel) and S63845 (lower panel) on the expression of granzyme b **(J)**, IFN-γ release **(K)** and the multifunctional CD8^+^ CAR-T cells (L) was determined after 6-h stimulation with 380 cells. Stronger effect of S63845 on CD19.CAR-T cell cytokine release function was observed, when compared to venetoclax. Three different individual donors have been analyzed. Each experiment was performed in triplicate. For statistical analysis a Tukey**’**s multiple comparisons test was used in GraphPad Prism 8.0. A *p* < 0.05 was considered to be statistically significant (*).

To determine whether combinatorial treatment with CD19.CAR-T cells and BH3 mimetics could produce superior antitumor effects, we cultured CD19.CAR-T cells with tumor cells in the presence of venetoclax or S63845. Combination of venetoclax with CD19.CAR-T cells showed a better antitumor activity compared with either tested alone (**Figure 4A**). The same effect, however, was not observed in combination of S63845 and CD19.CAR-T cells, showing greater cytotoxic activity than S63845 alone but a decrease of killing efficiency compared to CD19.CAR-T cells alone (**Figure 4B**).

To further explore the effect of venetoclax and S63845 on CD19.CAR-T cells in the simultaneous treatment system, we evaluated their effects not only on the quantity of CD19.CAR-T cells, but also on the quality of CD19.CAR-T cells in terms of expression of activation marker CD137 and degranulation marker CD107a, as well as the cytokine release and multifunctionality. The toxicity of BH3 mimetics on the quantity of CD19.CAR-T cells was observed for both BH3 mimetics. As illustrated in **Figure 4C**, venetoclax provoked a dose-dependent reduction of CD8^+^ CAR-T cells, with a trend toward gradual decrease in CD4^+^ CAR-T cells. S63845 had even stronger negative effects on both CD4^+^ and CD8^+^ CAR-T cells, especially at high concentration (**Figure 4D**). The frequency of Annexin V^+^ CD19.CAR-T cells was pronouncedly increased in this simultaneous treatment system for venetoclax (**Figure 4E**) and S63845 (**Figure 4F**), suggesting that BH3 mimetics induced apoptosis contributed to the reduction of CD19.CAR-T cells. Moreover, our data showed that the frequency of Bcl-2^+^CD19.CAR-T cells was elevated after administration of venetoclax (**Figure 4G**) and Mcl-1^+^CD19.CAR-T cells increased in the presence of S63845 (**Figure 4H**), resulting in an increased sensitivity of CD19.CAR-T cells to BH3 mimetics.

Interestingly, inverse effects of BH3 mimetics were seen for the quality of CD19.CAR-T cells. The expression of CD137 (**Figure 4I**) and CD107a (**Figure 4J**) on CD8^+^ CAR-T cells was significantly increased after stimulation by tumor cells in the presence of venetoclax or S63845. In addition, S63845 could markedly elevate the frequency of IFN-γ^+^CD8^+^ CAR-T cells (**Figure 4K**) and the multifunctional CD8^+^ CAR-T cells in terms of CD107a^+^IFN-γ^+^TNF-α^+^ cells (**Figure 4L**), but had no effect on other single- and double-cytokine releasing CD19.CAR-T cells (**Supplementary Figure 5**).

### Expansion and Killing Capacity of CD19.CAR-T Cells in Different Treatment Systems

**Figure 5:**
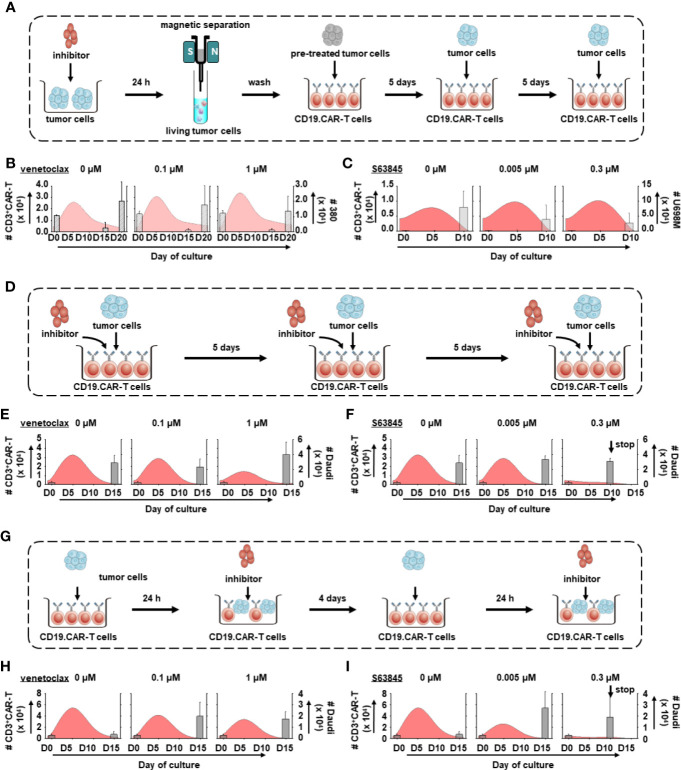
Dynamics of tumor cells and CD19.CAR-T cells in three different treatment systems. The schematic diagram of challenging assay of pre-treatment **(A)**, simultaneous treatment **(D)** and post-treatment system **(G)**. The influence of venetoclax **(B, E, H)** and S63845 **(C, F, I)** on dynamics of tumor cells (bar charts) and CD3^+^ CAR-T cells (curve charts) were determined by counting beads in pre-treatment **(B, C)**, simultaneous treatment **(E, F)** and post-treatment system **(H, I)**. The curve represents the mean value of the absolute count of CD3^+^ CAR-T cells. Our results show that pre-treated tumor cells could enhance the expansion of CD19.CAR-T cells but their expansion capacity could be hampered in simultaneous and post-treatment systems. Three different individual donors have been analyzed. Each experiment was performed in duplicates. The statistical analysis was performed using a paired-t test in IBM SPSS v22.

In an attempt to better understand the long-term effect of BH3 mimetics on CD19.CAR-T cells, three different *in vitro* treatment systems including pre- (**Figure 5A**), simultaneous (**Figure 5D**) and post-treatment (**Figure 5G**) have been established. The proliferative capacity of CD19.CAR-T cells, the number of challenging procedures, the number of residual tumor cells and the exhaustion status of CAR-T cells were investigated as key parameters.

BH3 mimetics low sensitive Daudi cells were chosen in simultaneous and post-treatment systems because (1) Daudi cells could stably stimulate CD19.CAR-T cells to proliferate (**Supplementary Figure 6**); (2) CD19.CAR-T cells could eliminate all Daudi cells within one round of challenging (**Supplementary Figure 6**), leading to a continuous challenge of CD19.CAR-T cells with fresh tumor cells to determine the long-term function of CAR-T cells; (3) use of Daudi cells could avoid the interference induced by the interaction between BH-3 mimetics and high-sensitive tumor cells, which might misrepresent the direct influence of BH-3 mimetics on CD19.CAR-T cells. It is different comparing to the pre-treatment system that focuses on the influence of BH-3 mimetics on tumor cells; (4) last but not least, since 380 cells are highly sensitive to venetoclax and U698M cells to S63845, dramatically reduced tumor cells may hamper the expansion of CAR-T cells leading to a failure of second challenge (data not shown).

**Figure 6:**
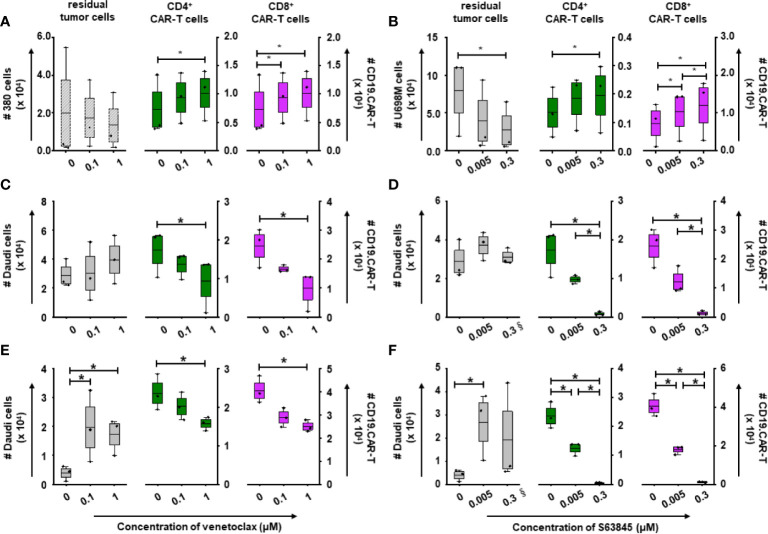
Killing efficiency and expansion of CD19.CAR-T cells in three different treatment systems. The residual tumor cells on the last day of challenge (day 15), as well as the expansion of CD4^+^ and CD8^+^ CAR-T cells on day 5 were determined by counting beads in pre-treatment **(A, B)**, simultaneous treatment **(C, D)** and post-treatment system **(E, F)**. The decreased number of residual tumor cells suggests that the long-term killing capacity of CD19.CAR-T cells could be maintained in the pre-treatment system, but not in the simultaneous and post-treatment systems. Pre-treated tumor cells could enhance the expansion of both CD4^+^ and CD8^+^CD19.CAR-T cells, while BH3 mimetics could significantly hinder their expansion. § means the number of the residual tumor cells on day 10. Three different individual donors have been analyzed. Each experiment was performed in duplicates. The statistical analysis was performed using a paired-t test in IBM SPSS v22. A *p* < 0.05 was considered to be statistically significant (*).

CD19.CAR-T cells rapidly proliferated upon the first tumor challenge, with a peak level of expansion occurring at day 5 (**Figures 5B, C, E, F, H, I**). BH3 mimetics pre-sensitized tumor cells facilitated the CAR-T cell proliferation (**Figures 5B, C**), with superior expansion of both CD4^+^ and CD8^+^ subsets (**Figures 6A, B**). However, the proliferation and expansion of CD19.CAR-T cells were apparently hampered by BH3 mimetics in a dose-dependent manner in both simultaneous (**Figures 5E, F, 6C, D**) and post-treatment systems (**Figures 5H, I, 6E, F**).

The maximum number of challenging procedures, which was determined by inability of CAR-T cells to expand and to eliminate tumor cells, varied among the different tumor cells (U698M cells: 3 times, Daudi cells: 4 times, and 380 cells: 5 times). Both BH3 mimetics did not reduce the number of challenging procedures (**Figures 5B, C, E, H**) with the exception of S63845 at highest concentration of 0.3 μM (**Figures 5F, I**), showing a failure of CD19.CAR-T cells in the second challenging procedure in simultaneous (**Figure 5F**) and post-treatment systems (**Figure 5I**).

Additionally, we observed a powerful reduction in the number of residual tumor cells at the end of culture in the pre-treatment system (**Figures 6A, B**), but not in simultaneous (**Figures 6C, D**) and post-treatment systems (**Figures 6E, F**).

### Persistence of CD19.CAR-T Cells Was Improved by Pre-treatment of Tumor Cells With BH3 Mimetics

**Figure 7:**
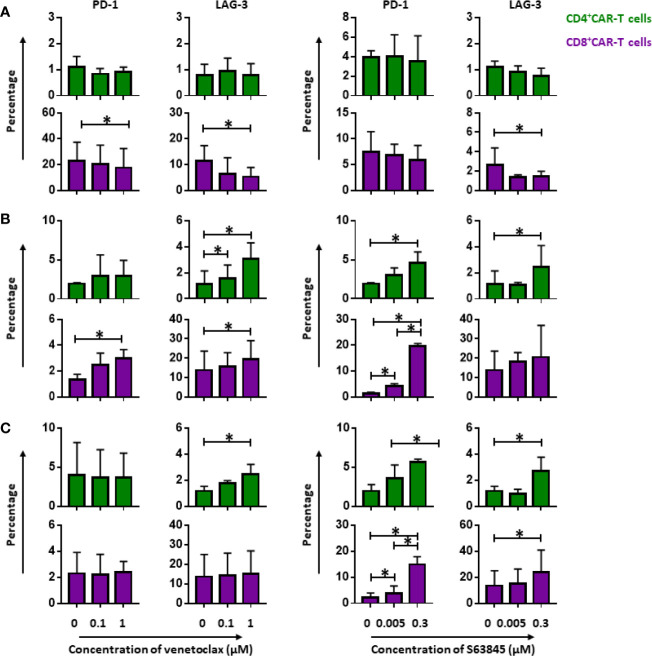
Persistence of CD19.CAR-T cells in three different treatment systems. The influence of venetoclax (left panel) and S63845 (right panel) on the expression of exhaustion markers, PD-1 and LAG-3, on CD4^+^ (upper panel) and CD8^+^ CAR-T cells (lower panel) in pre-treatment system **(A)**, simultaneous treatment system **(B)** and post-treatment system **(C)**. Persistence of CD19.CAR-T cells was improved in pre-treatment system, showing a decrease of exhaustion marker. A contrary result was observed in simultaneous and post-treatment system. Three different individual donors have been analyzed. Each experiment was performed in duplicates. The statistical analysis was performed using a paired-t test in IBM SPSS v22. A *p* < 0.05 was considered to be statistically significant (*).

To investigate the influence of BH3 mimetics on the persistence of CD19.CAR-T cells, the expression of inhibitory molecules PD-1 and LAG-3 on both CD4^+^ and CD8^+^ CAR-T cells were monitored during the repetitive tumor challenge in pre-,simultaneous and post-treatment systems. Interestingly, CD8^+^ CAR-T cells were not exhausted so strongly by pre-sensitized tumor cells compared to tumor cells without pretreatment. This was evidenced by minor expression of PD-1 and LAG-3 on CAR-T cells (**Figure 7A**). Besides this, pretreatment of tumor cells with venetoclax could ameliorate the exhaustion status of CD4^+^ CAR-T cells with reduction of PD-1 expression as well (**Figure 7A**).

In simultaneous (**Figure 7B**) and post-treatment (**Figure 7C**), however, we obtained contrary results concerning the effect of BH3 mimetics on CAR-T cell persistence. When compared to the group without BH3 mimetic treatment, both CD4^+^ and CD8^+^ CAR-T cells were more prone to exhaustion in the presence of BH3 mimetics (**Figures 7B, C**).

## Discussion

CD19.CAR-T cells have emerged as a potent therapeutic weapon against B cell hematologic malignancies, leading to an exponential increase of clinical trials (13). Multicenter trails with a larger number of patients have demonstrated the safety and efficacy of CD19.CAR-T cell therapy (14). Therefore, the FDA and EMA have approved two CD19.CAR-T cell products in USA and Europe. This has led to a paradigm shift of cancer therapy from chemotherapy followed by stem-cell transplantation to adoptive immunotherapy.

Although complete remission has been achieved in 60–90% of patients with B cell acute lymphoblastic leukemia (15–17), CD19.CAR-T cells are less effective for diffuse large B-cell lymphoma (40–50%) (17, 18) and CLL (15–30%) (19, 20). In CLL this might be due to the impaired CAR-T cell production (21), which highlights the need for combination therapies (22). One approach to improve the clinical outcome might be the combination of CAR-T cell therapy with BH3-mimetic drugs e.g. venetoclax. Importantly, our study could show that pre-sensitization of tumor cells with BH3 mimetics significantly strengthened the anti-tumor activity of CD19.CAR-T cells, and moreover enhanced the early expansion and long-term persistence of CD19.CAR-T cells *in vitro*.

A hallmark of cancer biology is resistance to cell death (23). Overexpression of anti-apoptotic Bcl-2 family proteins plays an important role in tumor resistance to apoptosis (24). Many hematologic malignancies including CLL and NHL highly express anti-apoptotic Bcl-2 proteins thus providing a survival advantage (25, 26). Notably, those proteins are even overexpressed in tumor stem cells that are responsible for tumorigenesis, early metastasis, relapse and chemoresistance (27). Accordingly, strategies to neutralize the anti-apoptotic Bcl-2 proteins thereby restoring apoptosis and eliminating tumor stem cells have led to the development of BH3 mimetics as therapeutic agents.

The BH3 mimetic compound venetoclax, a potent and selective Bcl-2 inhibitor, could mimic BH3-only proteins and bind to the hydrophobic groove of Bcl-2, thereby activating and oligomerizing BAK through displacing BAX and BIM from Bcl-2. Subsequently, cytochrome c is released from mitochondria and activates caspase 9, resulting in cell death (28, 29). Venetoclax has demonstrated promising preclinical activity against diverse hematological tumor cells *in vitro* and *in vivo* (30). Remarkably, subsequent clinical studies including phase I and phase II trials showed encouraging results that venetoclax induced high and durable responses in patients with r/r CLL, where an overall response rate of 79% and a complete remission rate of 20% were observed (31). Consequently, the FDA has approved venetoclax for use in patients with CLL as monotherapy after 2 lines or with del(17p), after one line in combination with rituximab (CLL MURANO) and first line in combination with obinutuzumab (CLL14). In NHL, phase I/II clinical trials have shown it to be safe and effective.

In addition, another BH3 mimetic compound S63845 has been developed, which specifically binds with high affinity to the BH3-binding groove of Mcl-1 (25), since 10% of cancers highly express Mcl-1 that not only mediates malignant cell survival and expansion in several primary tumors but also contributes to tumor resistance to various chemotherapeutic agents (28, 32). Previous studies demonstrated that S63845 could kill effectively Mcl-1 dependent tumor cells in a BAX-BAK-dependent fashion (25, 29).

In our study, the administration of these two BH3 mimetics as single agent to a panel of B cell leukemia and lymphoma cell lines showed differential activity. Both venetoclax and S63845 exerted selective cytotoxicity against the tumor cells which highly expressed Bcl-2 or Mcl-1 protein, but had limited efficacy in tumor cells with low expression of Bcl-2 or Mcl-1 consistent with previous studies (25, 33, 34). Therefore, our data support that the sensitivity of tumor cells to BH3 mimetics increases with up-regulating expression of Bcl-2 and Mcl-1. Notably, primary CLL cells are sensitive to apoptosis induction by both venetoclax and S63845.

To our knowledge, mono-therapy in cancer treatment could easily drive tumor cells resistant to the current therapy, resulting in aggressive relapse eventually. As reported, single-agent therapy with BH3 mimetics caused tumor cell resistance to treatment by up-regulation of non-targeted anti-apoptotic proteins and increased activation of PI3K/AKT/mTOR pathway, providing a rationale for a combinatorial therapy (35, 36). Hence, therapeutic strategies that could dual-hit tumor cells through different mechanisms are highly desirable.

CAR-T cells induce tumor cell apoptosis through different mechanisms than BH3 mimetics, including expression of cytolytic enzymes as perforin and granzyme, trimerization of Fas receptor by Fas ligand, release of effector cytokines, as well as secretion of extracellular vesicles (37, 38). Therefore, combination of CAR-T cells with BH3 mimetics might be an interesting strategy. In our combinatorial approaches, the pre-sensitization of tumor cells with BH3 mimetics followed by CAR-T cell therapy was superior to simultaneous and post-treatment strategies.

The simultaneous combination approach of venetoclax or S63845 with CD19.CAR-T cells showed conflicting results. The killing efficiency was significantly increased by the combination of venetoclax and CD19.CAR-T cells compared to the single-agent treatment. Similarly, a previous study reported that combinatorial therapy of CD19.CAR-T cells with ABT737, a multi-target inhibitor blocking Bcl-2, Bcl-xl and Bcl-w, showed a better anti-tumor activity than either tested individually (39). By contrast, no effect was seen when S63845 and CD19.CAR-T cells were simultaneously administered. The discrepancy between these BH3 mimetics might be explained by the individual susceptibility of CD19.CAR-T cells to each inhibitor, demonstrating more significant reduction of both CD4^+^ and CD8^+^ CAR-T cells by S63845 than by venetoclax.

Even though venetoclax and S63845 adversely affected the quantity of CD19.CAR-T cells, the quality of CD8^+^ CAR-T cells in terms of activation, production of granzyme and cytokine release was enhanced by both BH3 mimetics. An explanation for the activation of CAR-T cells shown by increased expression of CD137 and followed by elevation of CD107a and IFN-γ might be the up-regulated target antigen CD19 on tumor cells by BH3 mimetic treatment leading to an enhanced signal I of activation in CD19.CAR-T cells. As described, CD8^+^ T cells can up-regulate CD137 after activation more rapidly and to higher levels than CD4^+^ T cells (40, 41). This might provide an explanation for this phenomenon observed in CD8^+^ but not in CD4^+^ CAR-T cells.

For tumor eradication *in vivo*, CAR-T cells are required multiple rounds of killing and subsequently driven into differentiation and exhaustion, which is one of the major hurdles against effective tumor clearance by CAR-T cells (42). Thus, the capacity of CD19.CAR-T cells to repetitively kill tumor cells is merited a particular evaluation. Our data indicate that both BH3 mimetics, when simultaneously combined with CAR-T cells, not only impaired the repetitive tumor killing potential of CAR-T cells and the long-term cytotoxicity, but also hampered the early proliferation of both CD4^+^ and CD8^+^ CAR-T cells. Similar results were obtained for venetoclax and S63845 treatment after CAR-T cell therapy (post-treatment), showing a disability of expansion and multi-round killing of CD19.CAR-T cells. Those adverse effects by BH3 mimetics on CAR-T cells might be explained by direct induction of apoptosis, sensitization of CAR-T cells to BH3 mimetics *via* increasing levels of inhibitor targeting proteins, and inhibition of T cell proliferation by Bcl-2 up-regulation (9, 43).

Moreover, we could demonstrate that BH3 mimetics might induce CAR-T cell exhaustion. As known, persistent antigen exposure is the key factor that leads to T cell exhaustion (44). In our simultaneous treatment setting, the killing efficacy of CAR-T cell was compromised by BH3 mimetics resulting in an increased number of residual tumor cells, causing a continuous antigen exposure to CAR-T cells. Those exhausted CAR-T cells might be not effective in eradicating tumor cells anymore. Additionally, they might also contribute to tumor epitope escape leading to relapse (44). Thus, based on our data, venetoclax or S63845 inhibitor therapy applied simultaneously with CAR-T cells or after CAR-T cell therapy (post-treatment) is not recommended.

Notably, pre-treatment with BH3 mimetics followed by CAR-T cell therapy might be a promising strategy. Karlsson and colleagues reported that CAR-T cells exhibited greater cytotoxicity on ABT737 pre-sensitized tumor cells compared to the non-pre-sensitized cells (39). Consistent with this study, we could also demonstrate that CAR-T cells benefit from the pre-treatment of tumor cells with venetoclax or S63845, showing an enhancement of anti-tumor activity. This pre-sensitization phenomenon might be ascribed to the ‘priming effect’ by BH3 mimetics on tumor cells (9, 45) indicating that pre-treatment could not only increase the sensitivity of tumor cells to BH3 mimetics through up-regulation of target Bcl-2 and Mcl-1 expression, but also increase of susceptibility to apoptosis *via* up-regulation of expression of pro-apoptotic protein BAK.

Besides the “priming effect”, evidence for the correlation between the antigen expression and cytolytic activity of CAR-T cells has been demonstrated in previous studies as well as our data, suggesting a critical role of the density of target antigen for successful clearance of tumor cells by CAR-T cells (46, 47). Thus, beneficial effects on the potency of CD19.CAR-T cells from the pre-sensitization by BH3 mimetics might also depend on the increased expression level of CD19 antigen on tumor cells, as observed in our study.

Moreover, up-regulation of CD19 antigen on tumor cells could also facilitate the activation and exponential expansion of CD19.CAR-T cells that is highly associated with the clinical outcomes (3, 48). This is substantiated by our own data as well, showing a greater proliferative potential of CD19.CAR-T cells stimulated by pre-treated tumor cells. This superior expansion of CAR-T cells might partly explain the enhancement of the multi-round killing ability that may be attributed to the consequent reduction of exhausted CD19.CAR-T cells. Besides the influence on the tumor cells and CAR-T cells, as previously reported, pre-treatment could also suppress the activated T regulatory cells or even myeloid-derived suppressor cells, and moreover result in epitope spreading that could help counter immune escape (39, 49).

Therefore, the BH-3 mimetic pre-treatment of patients with B cell hematologic malignancies before CAR-T cell therapy might be an interesting strategy.

This combination will not only further reduce the tumor burden, but could also pre-sensitize the tumor cells becoming more sensitivity to CD19.CAR-T cells due to the up-regulation of CD19 and apoptotic proteins. Importantly, this pre-treatment strategy could improve the functionality of CD19.CAR-T cells in terms of expansion, persistence and long-term killing capacity.

In conclusion, a simultaneous approach of CAR-T cell therapy together with BH3 mimetics as well as BH3 mimetics after CAR-T cell therapy is not advised. However, a powerful new therapeutic approach presents the pre-sensitization with BH3 mimetics prior to CAR-T cell administration, which might lead to an efficient improvement of CAR-T cell therapy. This constitutes an exciting new strategy which will be validated in future *in vivo* studies and clinical trials.

## Data Availability Statement

The original contributions presented in the study are included in the article/**Supplementary Material**. Further inquiries can be directed to the corresponding author.

## Ethics Statement

The studies involving human participants were reviewed and approved by Ethics Committee of the University of Heidelberg (S-254/2016). The patients/participants provided their written informed consent to participate in this study.

## Author Contributions

AS, MS, LW, and MY designed the research project. MY performed the experiments. SW and WG collected and stored the samples. MY and LW acquired and analyzed the data. MS, AS, MY, LW, BN, TS, LS, M-LS, AH-K, JH, CK, and VK discussed the data and the organization of the manuscript. LW, AS, and MY wrote the manuscript. All authors critically reviewed the manuscript, MS, AS, CM-T, LZ, and PD edited the manuscript. AS, MS, and LW supervised the work. All authors contributed to the article and approved the submitted version.

## Conflict of Interest

MS received funding for collaborative research from Apogenix, Hexal and Novartis, travel grants from Hexal and Kite, received financial support for educational activities and conferences from bluebird bio, Kite and Novartis, is a board member for MSD and (co-)PI of clinical trials of MSD, GSK, Kite, and BMS, as well as a co-founder and shareholder of TolerogenixX Ltd. AS received travel grants from Hexal and Jazz Pharmaceuticals, research grant from Therakos/Mallinckrodt, and is a co-founder of TolerogenixX LtD. AS and LW are part- or full-time employers of TolerogenixX Ltd. LS was employed by Takeda Pharma Vertrieb GmbH & Co. KG.

The remaining authors declare that the research was conducted in the absence of any commercial or financial relationships that could be construed as a potential conflict of interest.

The handling editor declared a past collaboration with one of the authors TS.
