# Supplementary material for: Pre-sensitization of Malignant B Cells Through Venetoclax Significantly Improves the Cytotoxic Efficacy of CD19.CAR-T Cells
Source: Front Immunol. 2020 Dec 9;11:608167. doi: 10.3389/fimmu.2020.608167 (PMC7756123; doi:10.3389/fimmu.2020.608167)
Supplement: Supplementary file 1 [file DataSheet_1.doc]

## Supplementary Table

### Supplementary Table 1. Primer list.

| Mcl-1 | forward | AGAAAGCTGCATCGAACCAT |
| --- | --- | --- |
|  | reverse | CCAGCTCCTACTCCAGCAAC |
| Bcl-2 | forward | GCCTTCTTTGAGTTCGGTGG |
|  | reverse | GAAATCAAACAGAGGCCGCA |
| Bcl-xl | forward | GTAAACTGGGGTCGCATTGT |
|  | reverse | TGCTGCATTGTTCCCATAGA |
| GAPDH | forward | TGCACCACCAACTGCTTAGC |
|  | reverse | GGCATGGACTGTGGTCATGAG |

### Supplementary Table 2. Antibody list.

| **Antibody** | **Dye** | **Isotype** | **Clone** | **V_working_ (μl)** | **Company** | **Cat.No.** |
| --- | --- | --- | --- | --- | --- | --- |
| Bcl-2 | PE-CF594 | Mouse BALB/c IgG1,k | Bcl-2/100 | 1 | Biosciences | 563601 |
| Bcl-xl | PerCP | IgG2a | 2H12 | 1 | NOVUS | NBP2-34531PCP |
| CD10 | PE-Cy7 | Mouse IgG1, k | HI10a | 1 | Biolegend | 312214 |
| CD107a | FITC | Mouse BALB/c IgG1,k | H4A3 | 10 | Biosciences | 555800 |
| CD137 | FITC | Mouse IgG1, k | 4B4 | 1 | eBioscience | 11-1379-42 |
| CD19 | PerCP | Mouse IgG1, k | HIB19 | 1 | Biolegend | 302228 |
| CD27 | PE-Cy7 | Mouse IgG1, k | M-T271 | 1 | Biolegend | 356412 |
| CD3 | PE-Cy7 | Mouse IgG1, k | UCHT1 | 1 | Biolegend | 300420 |
| CD3 | BV510 | Mouse IgG2a, k | OKT3 | 1 | Biolegend | 317332 |
| CD3 | FITC | Mouse IgG1, k | UCHT1 | 1 | Biolegend | 300440 |
| CD4 | PE-CF594 | Mouse IgG1, k | RPA-T4 | 1 | Bioscience | 562281 |
| CD4 | APC | Mouse IgG1, k | SK3 | 1 | Biolegend | 344614 |
| CD69 | APC | Mouse IgG1, k | FN50 | 1 | Biolegend | 310910 |
| CD8 | BV510 | Mouse BALB/c IgG1,k | SK1 | 1 | Biosciences | 563919 |
| CD8 | APC | Mouse IgG1, k | SK1 | 1 | Biolegend | 344722 |
| CD8 | PerCP | Mouse IgG1, k | SK1 | 0.5 | Biolegend | 344708 |
| gt F(ab’)2 IgG | PE | Ig | --- | 0.5 | Dianova | 109-116-088 |
| IFN-γ | APC | Mouse IgG1, k | B27 | 1 | eBioscience | 554702 |
| LAG-3 | APC | Mouse IgG1, k | 7H2C65 | 1 | Biolegend | 369212 |
| Mcl-1 | Alexa Fluor 488 | Mouse IgG1, k | LVUBKM | 1 | eBioscience | 53-9047-42 |
| PD-1 | Alexa Fluor 488 | Mouse IgG1, k | EH12.2H7 | 1 | Biolegend | 329936 |
| Tim-3 | BV421 | Mouse IgG1, k | F38-2E2 | 1 | Biolegend | 345008 |
| TNF-α | BV421 | Mouse IgG1, k | MAb11 | 1 | eBioscience | 562783 |

Abbrevations: V_working_ = volume of the antibody per 100 μl cocktail solution; Cat.No. = catalogue number; PE = phycoerythrin; PE-Cy7 = phycoerythrin-Cyanin 7; BV = Brilliant Violet; PerCP = peridinin chlorophyll; APC = allophycocyanin; APC-Cy7 = allophycocyanin-Cyanin 7; IFN-γ = Interferon-γ; TNF-α = Tumor necrosis factor-α; FITC = Fluorescence isothiocyanate; Mcl-1 = myeloid cell leukemia 1; Bcl-2 = B-cell lymphoma 2; Bcl-xl = B-cell lymphoma-extra large; PD-1 = Programmed cell death protein 1; TIM-3 = T-cell immunoglobulin and mucin-domain containing-3; LAG-3 = Lymphocyte-activation gene 3.

## Figure legend

**Supplementary Figure 1. The optimal cell number for a 48-hr cultivation analyzed by CellTiter Glo assay.** The cell number in logarithmic growth phase was chosen.

Supplementary Figure 2. The quality evaluation of manufactured 3^rd^ generation CD19.CAR-T cells. A representative dot plot of the transduction efficiency of CD19.CAR T cells (A). Transduction efficiency of CD19.CAR-T cells on day 7 (n = 12) (B). Expansion fold of CD19.CAR-T cells (n = 12) on day 10 (C), which is calculated as the following formula: [cell number on day 10]/[cell number on day 4] × 100. The viability of CD19.CAR-T cells on day 10 (D). The potency of the 3^rd^ generation CD19.CAR-T cells against CD19^+^ leukemia/lymphoma cells was determined by Calcein AM assay (E). The E:T ratio was 30:1. The percentage of lysis was calculated as the following formula: [experimental release – spontaneous release]/[maximal release – spontaneous release] × 100. Three different individual donors have been analyzed. Each group had three replicates. The MFI of CD19 expression on leukemia/lymphoma cell lines (F). Three different individual donors have been analyzed. Each group had three replicates. The statistical analysis was performed using a Tukey's multiple comparisons test in GraphPad Prism 8.0. A *p* < 0.05 was considered to be statistically significant (*).

Supplementary Figure 3. Pre-treatment of Daudi cells by venetoclax or S63845 had no effect on the cytotoxic efficiency of CD19.CAR-T cells. The killing efficiency of CD19.CAR-T cells against venetoclax (A) and S63845 (B) pre-treated Daudi cells determined by Calcein AM assay. The percentage of lysis was calculated as followed: [experimental release – spontaneous release]/[maximum release – spontaneous release] × 100. The pre-treatment duration was 24-hr. Three different individual experiments have been analyzed. Each experiment was performed in triplicates. For statistical analysis, a Tukey's multiple comparisons test was used in GraphPad Prism 8.0. A *p* < 0.05 was considered to be statistically significant (*).

Supplementary Figure 4. Bcl-2 and CD19 have a co-expression in cells, but not Mcl-1 and CD19.

The co-expressions of Bcl-2 with CD19 (**A**) and Mcl-1 with CD19 (**B**) were predicted using GeneMANIA database. GeneMANIA identifies the most related genes to the input gene set using a guilt-by-association approach based on a large database of functional interaction networks from multiple organisms. Each relative gene can be traced to the source network used for making the prediction. Networks names describe the data source and are either came from the PubMed originated data source, or simply the name of the data source. For the co-expression network, the score could be the Pearson correlation coefficient between the expression profiles of the two genes. The score is a number ranging from zero (no interaction) to 1 (strong interaction).

Supplementary Figure 5. Venetoclax and S63845 had no influence on the single- and double-cytokine released CD8^+^ CAR-T cells. The percentage of cytokine released CD8^+^ CAR-T cells determined by flow cytometry after co-cultured with Daudi cells in the presence of either venetoclax (A) or S63845 (B) for 6-hr. Multifunctional analysis of CD107a, TNF-α, and IFN-γ expression in CD8^+^ CAR-T cells was conducted by using BD Diva software. Upper panel shows the single-cytokine released CAR-T cells and lower panel shows the double-cytokine released CAR-T cells. Data obtained from three independent experiments. Each experiment has duplicates. For statistical analysis, a Tukey's multiple comparisons test was used in GraphPad Prism 8.0. A *p* < 0.05 was considered to be statistically significant (*).

**Supplementary Figure 6. Optimal effector to target (E:T) ratios for repetitive challenging assay.**

380, Daudi and U698M cells were co-cultured with CAR-T cells at different E:T ratios. Tumor cells (bar chat) and CD3^+^ CAR-T cells (curve chats) were determined by counting beads in each day until the end of culture. The curve charts and bar chats represent mean ± SD from one experiment in triplicates.
