# Supplementary figures and images for: Pre-sensitization of Malignant B Cells Through Venetoclax Significantly Improves the Cytotoxic Efficacy of CD19.CAR-T Cells
Source: Front Immunol. 2020 Dec 9;11:608167. doi: 10.3389/fimmu.2020.608167 (PMC7756123; doi:10.3389/fimmu.2020.608167)

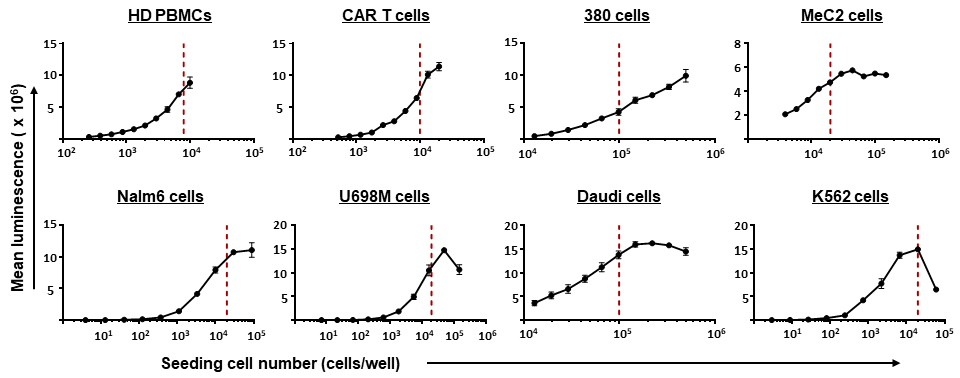

Supplement: Supplementary file 2 [file Image_1.tif]

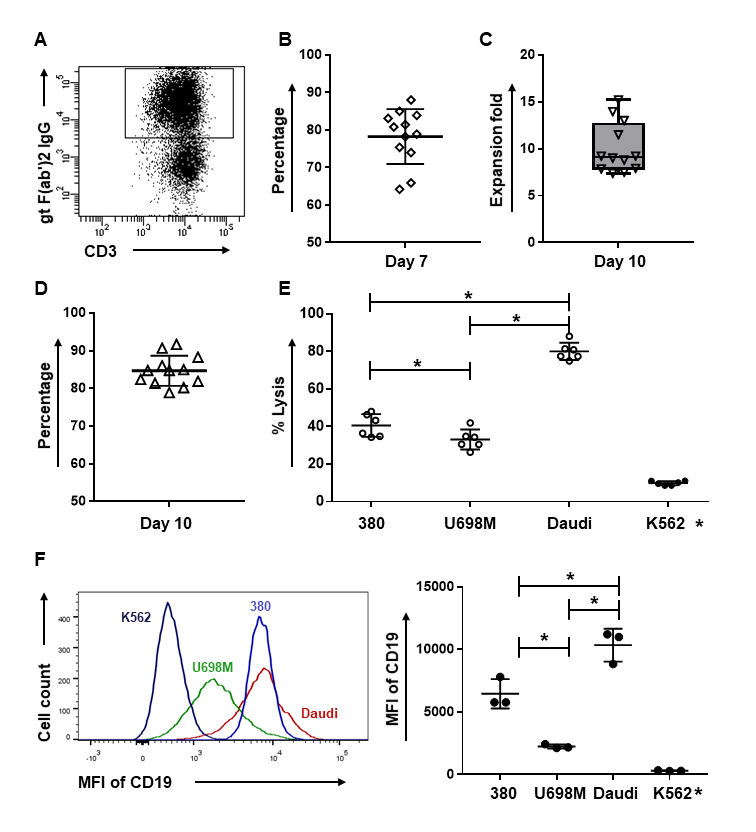

Supplement: Supplementary file 3 [file Image_2.tif]

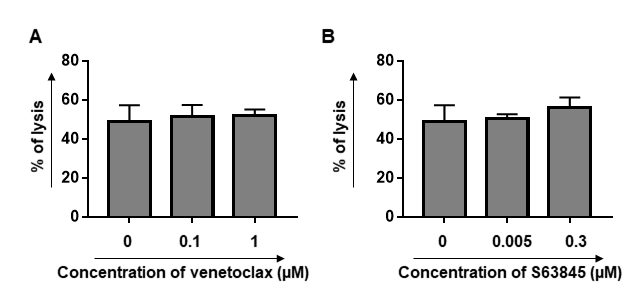

Supplement: Supplementary file 4 [file Image_3.tif]

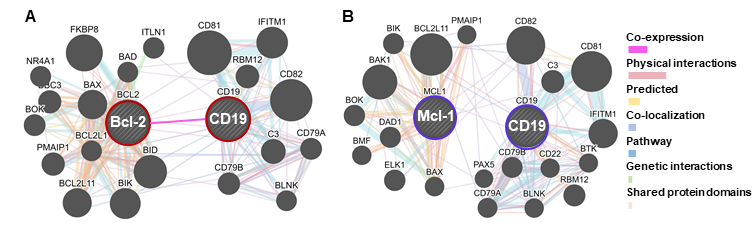

Supplement: Supplementary file 5 [file Image_4.tif]

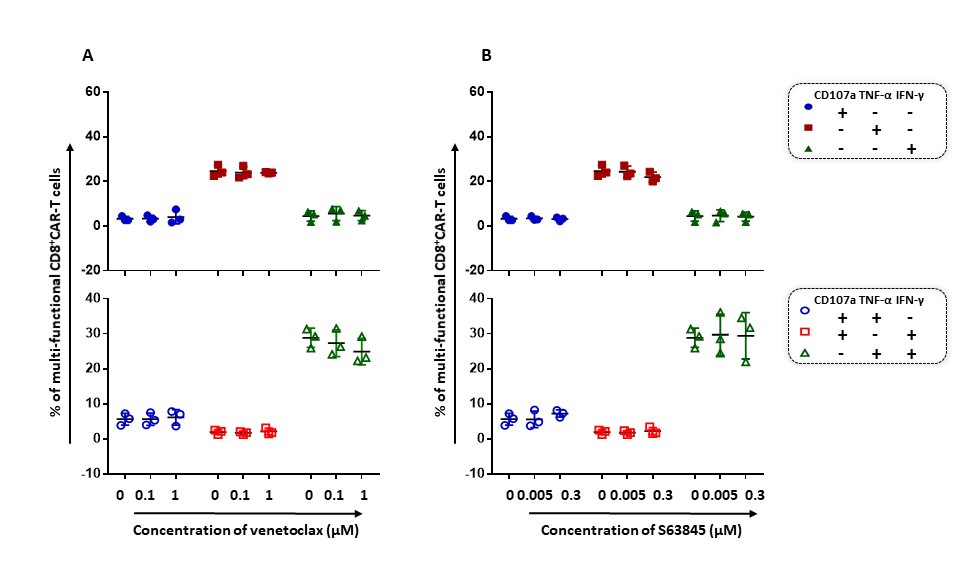

Supplement: Supplementary file 6 [file Image_5.tif]

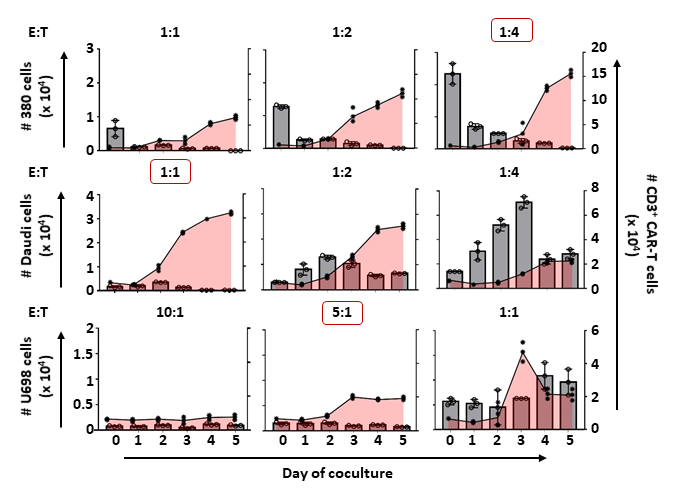

Supplement: Supplementary file 7 [file Image_6.tif]
